# Influence of different air CT numbers for IVDT on the dose distribution in TomoTherapy MVCT

**DOI:** 10.1002/acm2.13835

**Published:** 2022-10-31

**Authors:** Shogo Tsunemine, Shuichi Ozawa, Minoru Nakao, Hideharu Miura, Akito Saito, Daisuke Kawahara, Yasuhiko Onishi, Takashi Onishi, Taiki Hashiguchi, Yoshihisa Matsumoto, Tsutomu Maruta, Yuji Murakami, Yasushi Nagata

**Affiliations:** ^1^ Program of Medicine Doctoral Course Graduate School of Biomedical and Health Sciences, Hiroshima University Hiroshima Japan; ^2^ Radiation and Proton Therapy Center Shizuoka Cancer Center Shizuoka Japan; ^3^ Department of Radiology National Hospital Organization Himeji Medical Center Himeji Japan; ^4^ Department of Therapeutic Radiology National Hospital Organization Himeji Medical Center Himeji Japan; ^5^ Hiroshima High‐Precision Radiotherapy Cancer Center Hiroshima Japan; ^6^ Department of Radiation Oncology Graduate School of Biomedical and Health Sciences Hiroshima University Hiroshima Japan

**Keywords:** air CT number, ART, head‐and‐neck radiotherapy, helical TomoTherapy, IVDT, MVCT

## Abstract

This study aims to evaluate the effect of different air computed tomography (CT) numbers of the image value density table (IVDT) on the retrospective dose calculation of head‐and‐neck (HN) radiotherapy using TomoTherapy megavoltage CT (MVCT) images. The CT numbers of the inside and outside air and each tissue‐equivalent plug of the “Cheese” phantom were obtained from TomoTherapy MVCT. Two IVDTs with different air CT numbers were created and applied to MVCT images of the HN anthropomorphic phantom and recalculated by Planned Adaptive to verify dose distribution. We defined the recalculation dose with MVCT images using both inside and outside air of the IVDT as ${\mathrm{IVDT}}_{\mathrm{MVCT}}^{\mathrm{inair}}$ and ${\mathrm{IVDT}}_{\mathrm{MVCT}}^{\mathrm{outair}}$, respectively. Treatment planning doses calculated on kVCT images were compared with those calculated on MVCT images using two different IVDT tables, namely, ${\mathrm{IVDT}}_{\mathrm{MVCT}}^{\mathrm{inair}}$ and ${\mathrm{IVDT}}_{\mathrm{MVCT}}^{\mathrm{outair}}$. The difference between average MVCT numbers ±1 standard deviation on inside and outside air of the calibration phantom was 65 ± 36 HU. This difference in MVCT number of air exceeded the recommendation lung tolerance for dose calculation error of 2%. The dose differences between the planning target volume (PTV): *D*_98%_, *D*_50%_, *D*_2%_ and the organ at risk (OAR): *D*_max_, *D*_mean_ recalculated by ${\mathrm{IVDT}}_{\mathrm{MVCT}}^{\mathrm{inair}}$ and ${\mathrm{IVDT}}_{\mathrm{MVCT}}^{\mathrm{outair}}$ using MVCT images were a maximum of 0.7% and 1.2%, respectively. Recalculated doses to the PTV and OAR with MVCT showed that ${\mathrm{IVDT}}_{\mathrm{MVCT}}^{\mathrm{outair}}$ was 0.5%–0.7% closer to the kVCT treatment planning dose than ${\mathrm{IVDT}}_{\mathrm{MVCT}}^{\mathrm{inair}}$. This study showed that ${\mathrm{IVDT}}_{\mathrm{MVCT}}^{\mathrm{outair}}$ was more accurate than ${\mathrm{IVDT}}_{\mathrm{MVCT}}^{\mathrm{inair}}$ in recalculating the dose HN cases of MVCT using TomoTherapy.

## 1 INTRODUCTION

Adaptive radiotherapy (ART) has been widely used for the modification of a treatment plan in recent years.^1^ In TomoTherapy, megavoltage‐computed tomography (MVCT) imaging is performed before treatment. MVCT images are used for treatment position matching and confirmation of the anatomical changes that occur during treatment. MVCT images are used for ART to assess the planning target volume (PTV) and organ at risk (OAR).^2^ The initial treatment dose may not be achieved due to weight loss and tumor shrinkage during treatment are common in head‐and‐neck (HN) radiotherapy, and the need for retreatment planning has been reported in many clinical cases.^1^, ^3^, ^4^

ART planning is performed on MVCT images using image value density table (IVDT) tables created from MVCT number to mass density (MD) calibration. These results will be used to predict the radiation toxicity during treatment. Therefore, it is necessary to use the correct IVDT for accurate dose calculations.^5^ To create IVDT, an MVCT scan of the calibration phantom with tissue‐equivalent plugs are obtained, and MVCT numbers to MD can be established.^6^ There is little variation between computed tomography (CT) numbers for air, within and outside the calibration phantom, in kVCT.^7^ IVDT can be created with either value that may not make wide difference in kVCT dose calculations is used. In TomoTherapy, the air dose outside the body contour is also calculated, and the TomoTherapy white paper recommends using the MVCT number of air outside the calibration phantom to create the IVDT.^8^ In the case of IVDT, which uses the inside air MVCT number that causes the presence of air holes, there may also be an effect on the consistency between the results of the initial treatment planning with kVCT and the in‐treatment dose calculation with MVCT. To the best of our knowledge, the effect of different MVCT numbers of IVDT air on dose distribution calculations has not been reported. It is expected that MVCT number of air difference may affect the dose distribution calculation in the HN region, which is useful for ART.

The present study focused on HN radiotherapy, where dose calculation for ART using IVDT plays an important role. We investigated the influence of different air MVCT number of IVDT on ART dose calculations using an anthropomorphic phantom. In this study, IVDTs with different air MVCT numbers were used to verify the following two points: The first was to verify the change in dose calculation results owing to different MVCT numbers of air. The second was to confirm the consistency of the dose calculation results between the planning kVCT and MVCT. We propose to discuss the handling of air MVCT numbers in IVDT creation to perform highly accurate ART in TomoTherapy.

## 2 METHODS AND MATERIALS

### 2.1 ${\mathrm{IVDT}\;}_{\mathrm{MVCT}}^{\mathrm{outair}}$ and ${\mathrm{IVDT}\;}_{\mathrm{MVCT}}^{\mathrm{inair}}$ for MVCT

**FIGURE 1:**
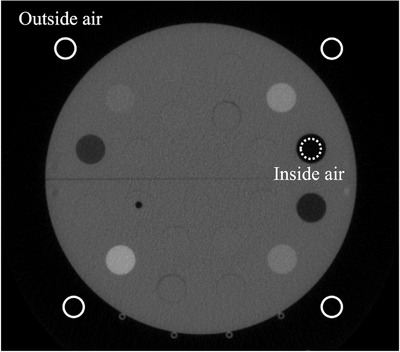
Measurement ROI for outside air (solid line) and inside air (dashed line) on calibration phantom

Calibration phantom images were acquired using TomoTherapy MVCT to create IVDTs using inside and outside air MVCT numbers. The scan condition of TomoTherapy equipped with MVCT can change only pitch ratio for fine, normal, coarse. This pitch ratio has little effect on the MVCT numbers,^5^ the pitch used normal mode in this study. We defined the recalculation dose with MVCT using inside air of the IVDT and outside air of the IVDT as ${\mathrm{IVDT}\;}_{\mathrm{MVCT}}^{\mathrm{inair}}$ and ${\mathrm{IVDT}\;}_{\mathrm{MVCT}\;}^{\mathrm{outair}}$, respectively. To create the IVDTs, a cheese phantom (Accuray, Inc., Madison, WI, USA) and Gammex tissue‐equivalent plugs (Middleton, WI, USA) of 0.001–1.822 g/cm^3^ were used. The average MVCT number of the inner region (diameter = 20 mm) of each plug was used to create the IVDTs. The MVCT number was obtained using ImageJ software version 1.51. Figure 1 shows the MVCT number of air acquired inside and outside the calibration phantom. Using the MVCT number obtained from each tissue‐equivalent plug and the air inside and outside the calibration phantom, ${\mathrm{IVDT}\;}_{\mathrm{MVCT}}^{\mathrm{inair}}$ and ${\mathrm{IVDT}\;}_{\mathrm{MVCT}}^{\mathrm{outair}}$ were created and used in TomoTherapy Planning Station 5.11 (Accuray Incorporated, Sunnyvale, CA, USA).

### 2.2 Verification treatment plan

**FIGURE 2:**
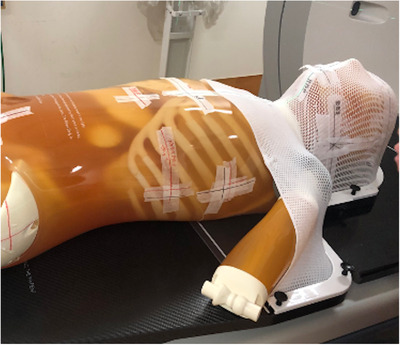
A setup of an anthropomorphic head‐and‐neck phantom

**FIGURE 3:**
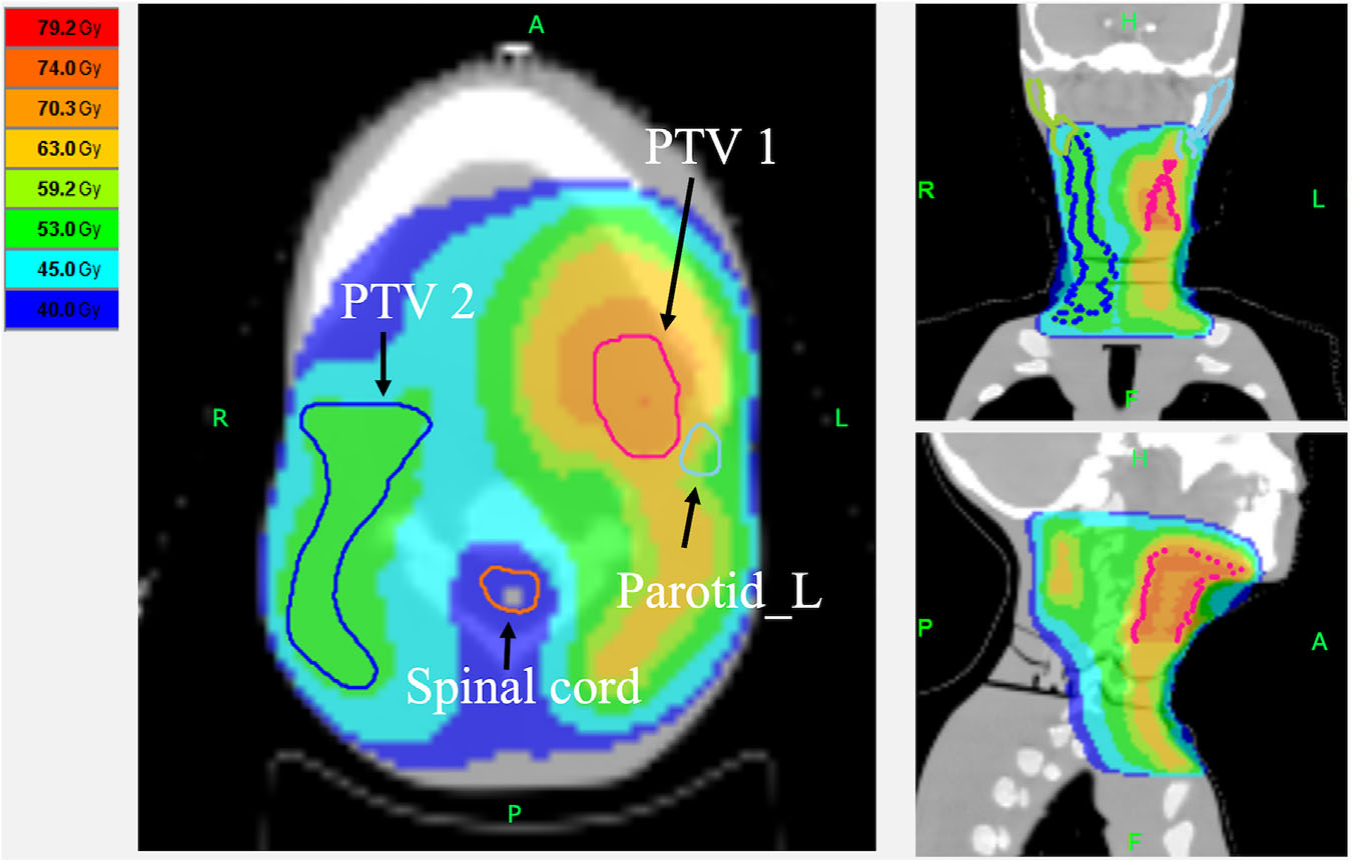
An example of dose distribution in head‐and‐neck case using anthropomorphic phantom

For validation, an anthropomorphic phantom PBU‐60 (Kyoto Kagaku, Kyoto, Japan), including a full‐scale artificial skeleton and soft tissue, was used. The phantom weighs 50 kg and has a length of 165 cm, soft‐tissue density of 1.061 g/cm^3^, and relative electron density of 0.975. The kVCT images were acquired using Aquilion 16 (Canon Medical System, Tokyo, Japan) with a tube potential of 120 kV, current of 300 mA, field of view (FOV) of 500 mm, and CT slice thickness of 2 mm. The phantom was immobilized with an HN shell (CIVCO Medical Solutions, Orange City, IA, USA) to minimize the setup errors (Figure 2). Contours of body structure and simulated tumors were created with MIM maestro (MIM Software Inc., Cleveland, OH, USA). The HN treatment plan was planned to use a simultaneous integrated boost‐IMRT technique. PTV was defined by adding a 5 mm margin around the simulated tumor. The prescribed dose was 70 Gy in 35 fractions. The prescribed dose to cover 95% of the PTV was optimized. The treatment plan parameters were set to a pitch of 2.0, field width of 2.512 cm, and modulation factor of 0.215. Dose calculations were performed using the superposition algorithm with a grid size of 1.95 mm × 1.95 mm (Figure 3). The CT number‐to‐MD calibration was performed using IVDT_kVCT_. The IVDT_kVCT_ was created with a calibration phantom taken under the same conditions as the kVCT in the verification HN treatment plan.

### 2.3 Dose calculation for MVCT image

**FIGURE 4:**
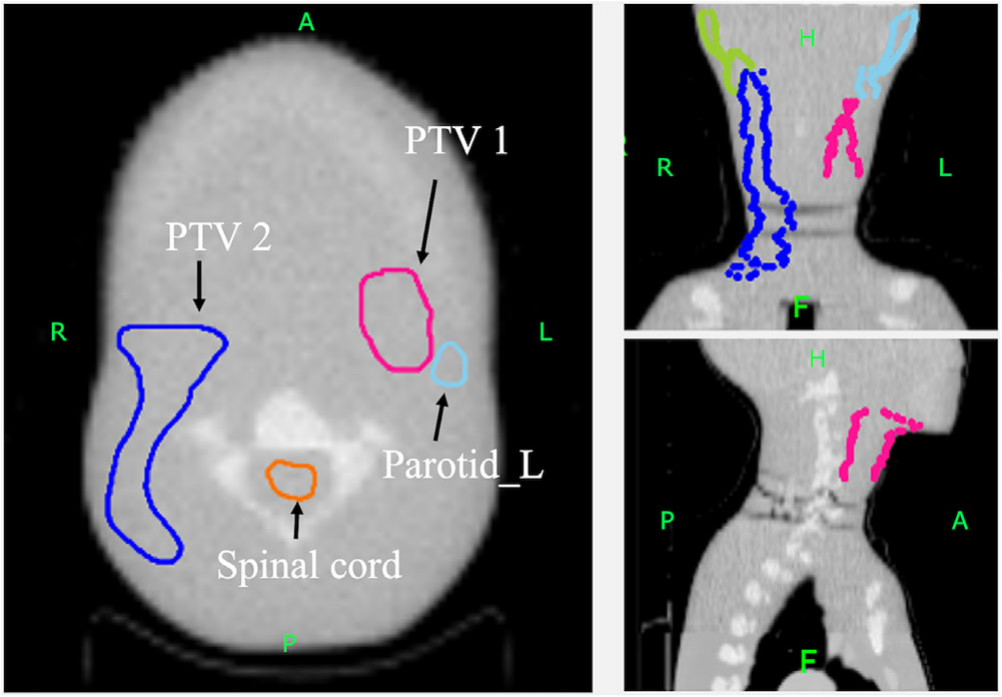
Three‐dimensional planes for anthropomorphic head‐and‐neck phantom on megavoltage computed tomography (MVCT) images

MVCT images of the phantom were acquired using the TomoTherapy HD System (Accuray Incorporated, Sunnyvale CA, USA) with a tube potential of 3.5 MV, FOV of 400 mm, and slice thickness of 2 mm (Figure 4). Dose calculation was performed using the Planned Adaptive Software (Accuray Incorporated, Sunnyvale CA, USA). The dose distribution on the MVCT images was calculated using different IVDT curves for the air MVCT number. Dose calculations were performed using the superposition algorithm with a grid size of 1.95 mm × 1.95 mm (Figure 3). A dose–volume histogram (DVH) was used for evaluation. The DVH evaluation points were *D*_98%_, *D*_2%_ for PTV, and *D*_max_, *D*_mean_ for OAR. MVCT images were used for ${\mathrm{IVDT}\;}_{\mathrm{MVCT}}^{\mathrm{outair}}$ and ${\mathrm{IVDT}\;}_{\mathrm{MVCT}}^{\mathrm{inair}}$, and the evaluation by dose recalculation comparison was performed using the following equation:

(1) $$\Delta D_{\alpha }=\frac{\left({\mathrm{ART}\;\mathrm{dose}}_{\mathrm{IVDTinair}}^{\mathrm{MVCT}}-{\mathrm{ART}\;\mathrm{dose}}_{\mathrm{IVDToutair}}^{\mathrm{MVCT}}\right)}{{\mathrm{ART}\;\mathrm{dose}}_{\mathrm{IVDToutair}}^{\mathrm{MVCT}}}\;\left(\%\right)$$

where ${\mathrm{ART}\;\mathrm{dose}}_{\mathrm{IVDTinair}}^{\mathrm{MVCT}}\;$and ${\mathrm{ART}\;\mathrm{dose}}_{\mathrm{IVDToutair}}^{\mathrm{MVCT}}\;$show the recalculated results using ${\mathrm{IVDT}\;}_{\mathrm{MVCT}}^{\mathrm{inair}}\;$and ${\mathrm{IVDT}\;}_{\mathrm{MVCT}}^{\mathrm{outair}}$, respectively.

A comparison of recalculated doses using MVCT images with ${\mathrm{IVDT}\;}_{\mathrm{MVCT}}^{\mathrm{inair}}$ and ${\mathrm{IVDT}\;}_{\mathrm{MVCT}}^{\mathrm{outair}}$ based on the DVH of the kVCT verification treatment plan was performed using the following equation:

(2) $$\;\Delta D_{\beta }=\frac{\left({\mathrm{ART}\;\mathrm{dose}}_{\mathrm{IVDTmodify}}^{\mathrm{MVCT}}\;-\mathrm{Plan}\;\mathrm{dos}e^{\mathrm{kVCT}}\right)}{\mathrm{Plan}\;\mathrm{dos}e^{\mathrm{kVCT}}}\;\left(\%\right)$$

where ${\mathrm{ART}\;\mathrm{dose}}_{\mathrm{IVDTmodify}}^{\mathrm{MVCT}}\;$indicates the result of re‐dose calculation by MVCT using ${\mathrm{IVDT}\;}_{\mathrm{MVCT}}^{\mathrm{inair}}\;$and ${\mathrm{IVDT}\;}_{\mathrm{MVCT}}^{\mathrm{outair}}$, respectively, and Plan dose^kVCT^ indicates the kVCT verification treatment plan dose.

## 3 RESULTS

### 3.1 ${\mathrm{IVDT}\;}_{\mathrm{MVCT}}^{\mathrm{inair}}$ and ${\mathrm{IVDT}\;}_{\mathrm{MVCT}}^{\mathrm{outair}}$　 for MVCT

**FIGURE 5:**
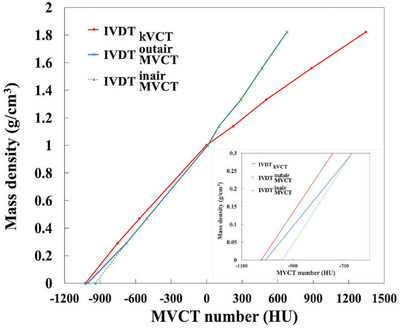
Comparison between IVDT_kVCT_ and IVDT_MVCT_. IVDT_kVCT_ (red solid line); ${\mathrm{IVDT}\;}_{\mathrm{MVCT}}^{\mathrm{inair}}$ computer tomography (CT) number of air was −940 HU (green dashed line); ${\mathrm{IVDT}\;}_{\mathrm{MVCT}}^{\mathrm{outair}}\;$CT number of air was −1006 HU (blue solid line).

${\mathrm{IVDT}\;}_{\mathrm{MVCT}}^{\mathrm{inair}}\;$and ${\mathrm{IVDT}\;}_{\mathrm{MVCT}}^{\mathrm{outair}}\;$created with the MVCT number of the air and each tissue plug inside and outside the calibration phantom and the IVDT_kVCT_ used in the verification treatment plan are shown in Figure 5. Average of the inside and outside on calibration phantom MVCT number of air variations (±1 standard deviation) were −940 ± 31 and −1006 ± 19 HU, respectively. The inside air MVCT number was 65 ± 36 HU higher than the outside air MVCT number.

### 3.2 Comparison between ${\mathrm{IVDT}\;}_{\mathrm{MVCT}}^{\mathrm{inair}}$ and ${\mathrm{IVDT}\;}_{\mathrm{MVCT}}^{\mathrm{outair}}$ dose calculations

**FIGURE 6:**
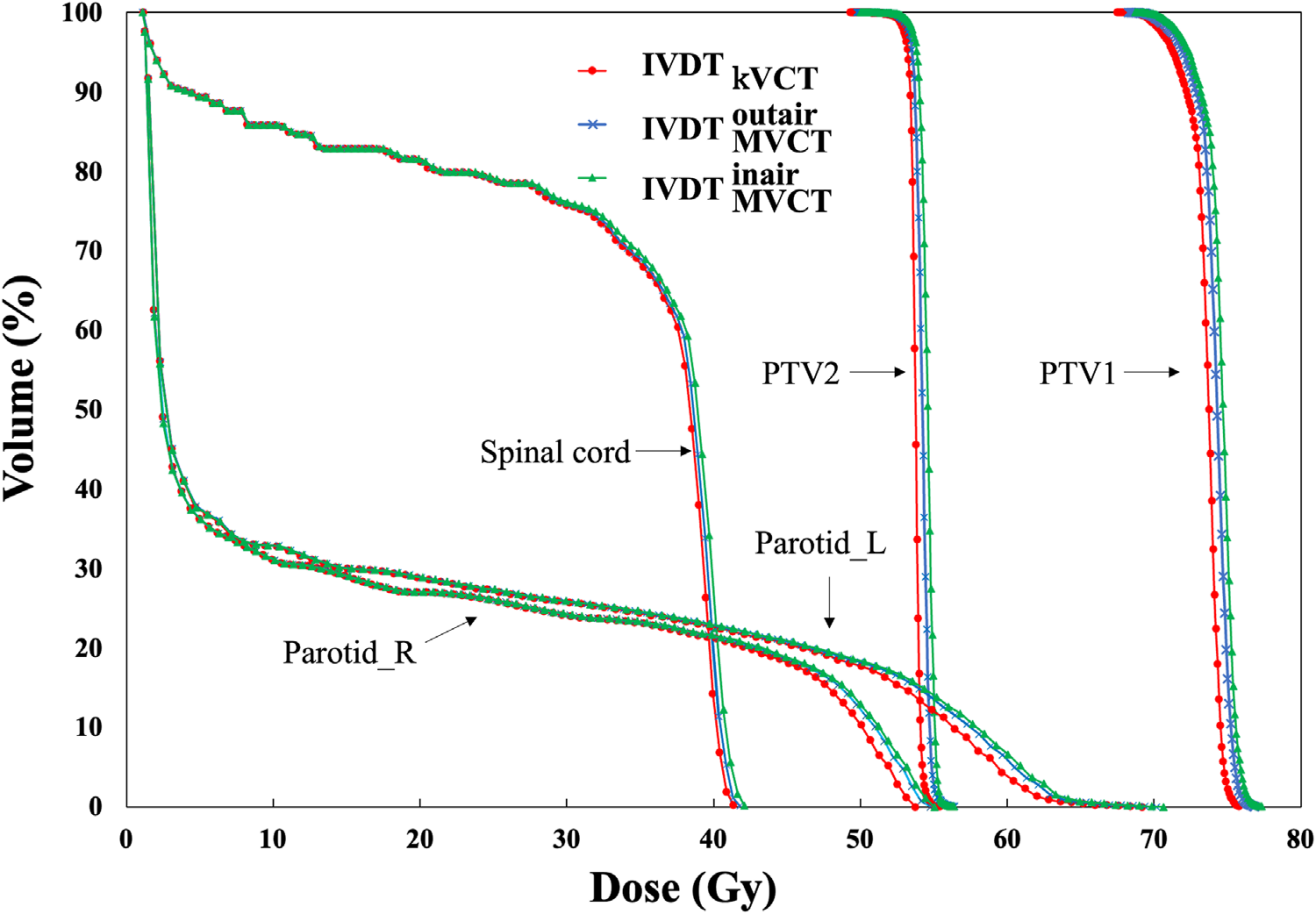
Comparison between dose–volume histograms (DVHs) of kVCT planning and recalculated DVHs using megavoltage computed tomography (MVCT). kVCT plan (red solid line), MVCT ${\mathrm{IVDT}\;}_{\mathrm{MVCT}}^{\mathrm{inair}}$ (blue solid line), and MVCT ${\mathrm{IVDT}\;}_{\mathrm{MVCT}}^{\mathrm{outair}}$ (green solid lines)

**FIGURE 7:**
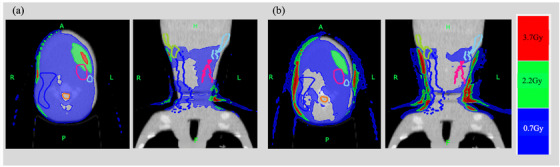
Difference between the initial kVCT planning dose distribution for head and neck. Radiotherapy planning and the dose distributions calculated by (a) ${\mathrm{IVDT}\;}_{\mathrm{MVCT}}^{\mathrm{inair}}$ and (b) ${\mathrm{IVDT}\;}_{\mathrm{MVCT}}^{\mathrm{outair}}$, respectively

Table 1 shows the comparison results of the DVHs’ dosimetry indices calculated by ${\mathrm{IVDT}}_{\mathrm{MVCT}}^{\mathrm{inair}}$ and ${\mathrm{IVDT}}_{\mathrm{MVCT}}^{\mathrm{outair}}\;$using MVCT images of the HN region. Figure 6 shows a comparison of the recalculated DVHs for the HN treatment plan. The results show that, for all DVHs, ${\mathrm{IVDT}\;}_{\mathrm{MVCT}}^{\mathrm{outair}}\;$shows a curve closer to the kVCT plan than ${\mathrm{IVDT}\;}_{\mathrm{MVCT}}^{\mathrm{inair}}$. Figure 7 shows a comparison of ${\mathrm{IVDT}\;}_{\mathrm{MVCT}}^{\mathrm{inair}}\;$and ${\mathrm{IVDT}\;}_{\mathrm{MVCT}}^{\mathrm{outair}}$. The dose difference between the dose distribution calculated by each and the dose distribution of the kVCT treatment plan is shown. In the HN region, ${\mathrm{IVDT}\;}_{\mathrm{MVCT}}^{\mathrm{outair}}\;$had a difference of 3.7 Gy outside the body contour; however, within the body contour, the difference from the kVCT treatment plan was less than that of ${\mathrm{IVDT}\;}_{\mathrm{MVCT}}^{\mathrm{inair}}$. Table 2 shows the comparison results of each dose index of DVHs between the kVCT planning dose and the dose results recalculated using MVCT images with ${\mathrm{IVDT}\;}_{\mathrm{MVCT}}^{\mathrm{inair}}$ and ${\mathrm{IVDT}\;}_{\mathrm{MVCT}}^{\mathrm{outair}}$. ${\mathrm{IVDT}\;}_{\mathrm{MVCT}}^{\mathrm{inair}}$ and ${\mathrm{IVDT}\;}_{\mathrm{MVCT}}^{\mathrm{outair}}$ differed kVCT planning dose were 1.1%–2.4% and 0.9%–1.9%, respectively. In the HN results, compared to ${\mathrm{IVDT}\;}_{\mathrm{MVCT}}^{\mathrm{inair}}$, ${\mathrm{IVDT}\;}_{\mathrm{MVCT}}^{\mathrm{outair}}\;$was 0.5%–0.7% closer to the kVCT planning dose.

**TABLE 1:** Comparison between treatment plans using ${\mathrm{IVDT}\;}_{\mathrm{MVCT}}^{\mathrm{outair}}$ and ${\mathrm{IVDT}\;}_{\mathrm{MVCT}}^{\mathrm{inair}}$

HN radiotherapy		MVCT—adaptive dose (Gy)		
Name		${\mathrm{IVDT}\;}_{\mathrm{MVCT}}^{\mathrm{inair}}$	${\mathrm{IVDT}\;}_{\mathrm{MVCT}}^{\mathrm{outair}}$	Δ*D_α_* (%)
PTV1	*D*_98%_	71.4	71.0	0.6
	*D*_50%_	74.7	74.3	0.5
	*D*_2%_	76.2	75.8	0.5
PTV2	*D*_98%_	53.2	52.9	0.6
	*D*_50%_	54.6	54.2	0.7
	*D*_2%_	55.1	54.4	1.2
Spinal cord	*D*_max_	42.3	42.0	0.7
Parotid_R	*D*_max_	55.0	54.8	0.5
	*D*_mean_	28.1	28.0	0.5
Parotid_L	*D*_max_	70.6	70.2	0.6
	*D*_mean_	36.1	35.8	0.6

Abbreviations: *D*_x%_, dose received by ≥*x*% volume; HN, head and neck; MVCT, megavoltage computed tomography;PTV, planning target volume.

**TABLE 2:** Dose calculation and dose difference from treatment plan with different image value density tables (IVDTs) air in head‐and‐neck (HN) case

HN radiotherapy		kVCT‐planning dose (Gy)	MVCT—adaptive dose (Gy)	Δ*D_β_* (%)		
Name		IVDT_kVCT_	${\mathrm{IVDT}\;}_{\mathrm{MVCT}}^{\mathrm{inair}}$	${\mathrm{IVDT}\;}_{\mathrm{MVCT}}^{\mathrm{outair}}$	${\mathrm{IVDT}\;}_{\mathrm{MVCT}\;}^{\mathrm{inair}}$	${\mathrm{IVDT}\;}_{\mathrm{MVCT}}^{\mathrm{outair}}$
PTV1	*D*_98%_	70.4	71.4	71.0	1.5	0.9
	*D*_50%_	73.7	74.7	74.3	1.3	0.8
	*D*_2%_	75.0	76.2	75.8	1.6	1.0
PTV2	*D*_98%_	52.9	53.5	53.2	1.1	0.6
	*D*_50%_	53.8	54.6	54.2	1.5	0.8
	*D*_2%_	54.4	55.3	55.1	1.7	1.2
Spinal cord	*D*_max_	41.5	42.3	42.0	1.8	1.1
Parotid_R	*D*_max_	53.7	55.0	54.8	2.4	1.9
	*D*_mean_	27.5	28.1	28.0	2.4	1.9
Parotid_L	*D*_max_	69.2	70.6	70.2	2.1	1.5
	*D*_mean_	35.3	36.1	35.8	2.0	1.4

Abbreviations: *D*_x%_, dose received by ≥*x*% volume; MVCT, megavoltage computed tomography;PTV, planning target volume.

## 4 DISCUSSION

In MVCT, the difference in MVCT number between inside and outside of the calibration phantom was 65 ± 36 HU. The variation in MVCT number ±50 HU for lung‐like materials is dose difference within 2%.^6^ This difference of MVCT number estimated from previous study exceeded dose difference 2%. The ${\mathrm{IVDT}\;}_{\mathrm{MVCT}}^{\mathrm{inair}}$ parotid results of this study exceeded a dose difference of 2% (Table 2). This value was close to the estimated value. Moreover, the difference in the average kVCT number between inside and outside of the phantom has been reported to be 3–13 HU for multi‐manufacturers’ kVCT devices [7]. Therefore, the difference in kVCT numbers between inside and outside air is one‐fifth of that of the MVCT number and can be considered clinically negligible. The difference in MVCT number of air inside and outside the phantom may be associated with the CT image reconstruction algorithm and beam hardening correction. The CT image reconstruction algorithms have been reported to result in maximum 56 and 116 HU changes for water and near‐bone densities, respectively.^9^ Additionally, TomoTherapy MVCT has a stronger capping artifact than the simulated kVCT system.^10^ The capping artifact is center ROI appears brighter than the periphery ROIs. In the beam hardening correction of kVCT, the center and outer air CT numbers are corrected to close values.^11^ On the other hand, MVCT image is considered to have a change in the air MVCT numbers inside and outside the phantom due to the small beam hardening correction effect. Hence, the MVCT may have resulted in a difference in the air MVCT number inside and outside the phantom.

In TomoTherapy, the calculation of PTV and OAR dose distributions using daily MVCT images is important for determining to modify the treatment plan. The HN regions where MVCT images were used, there was a 0.5%–1.2% impact depending on the different MVCT numbers of the IVDT air used for dose recalculation (Table 1). The results of the kVCT planning dose and the MVCT dose calculation for ${\mathrm{IVDT}\;}_{\mathrm{MVCT}}^{\mathrm{outair}}$ were within 2% of the dose results (Table 2), similar to previous reports [5]. The DVH and dosimetry indices showed that ${\mathrm{IVDT}\;}_{\mathrm{MVCT}}^{\mathrm{outair}}$ was closer to the kVCT planning dose than ${\mathrm{IVDT}\;}_{\mathrm{MVCT}}^{\mathrm{inair}}$ (Figure 7, Table 2). The body contour of the HN was smaller than that of the others, and the percentage of air outside the body contour within the FOV of 400 mm of the MVCT image was larger. The CT number of air in the IVDT_kVCT_ was −1023 HU; ${\mathrm{IVDT}\;}_{\mathrm{MVCT}}^{\mathrm{outair}}$ and ${\mathrm{IVDT}\;}_{\mathrm{MVCT}}^{\mathrm{inair}}$ were −1006 and −940 HU, respectively (Figure 5). The curve of ${\mathrm{IVDT}\;}_{\mathrm{MVCT}}^{\mathrm{outair}}$ is closer to IVDT_kVCT_; therefore, the density converted from the CT number by the IVDT is closer to the kVCT planned dose in ${\mathrm{IVDT}\;}_{\mathrm{MVCT}}^{\mathrm{outair}}$. We believe that the ${\mathrm{IVDT}\;}_{\mathrm{MVCT}}^{\mathrm{outair}}$ is more accurate for dose calculation in the HN region, and the percentage of air outside the body contour is large. As shown in Figure 5, the only difference in the two IVDTs is the CT number of 0.001 g/cm^3^, which is the same value for densities higher than −679 HU and 0.29 g/cm^3^. In Figure 7, the dose in the phantom differs between the initial treatment plan and the recalculation. This may be related to the difference in the calculated results of air attenuation in the beam path from IVDT.

Tumor shrinkage during treatment has also been reported to occur in the treatment of lung cancer.^12^ Mean lung dose greater than 20 Gy is associated with an increased risk of radiation pneumonitis^13^ and is an effective site for ART. However, it has been reported that lung density decreases from 0.25 to 0.37 g/cm^3^ in the normal lung to 0.11 g/cm^3^ in emphysema.^14^ Because the histogram of CT numbers varies widely from clinical case, it may not be possible to say whether ${\mathrm{IVDT}\;}_{\mathrm{MVCT}}^{\mathrm{inair}}$ or ${\mathrm{IVDT}\;}_{\mathrm{MVCT}}^{\mathrm{outair}}\;$is preferable. The dose difference exceeds 3.7 Gy only in the air layer between the HN shell and the body surface (Figure 7). This region contains more MVCT number below −679 HU. Therefore, for ROIs that contain many areas below −679 HU are in the vicinity, using in air MVCT number rather than out air may provide results closer to the initial treatment plan.

In this study, the superposition method was used as the calculation algorithm for the Planned Adaptive TomoTherapy HD system. A difference of 2.0%–3.0% in the accuracy of dose calculation in the superposition method compared with the Monte Carlo method has been reported.^14^ Therefore, a different trend can be observed in Monte Carlo calculations. In the chest region, lung density varies with lung pathology.^15^ In addition, the lungs are large within the human body, so the effect of the air MVCT number of the IVDT is expected to be significant. Therefore, whether ${\mathrm{IVDT}\;}_{\mathrm{MVCT}\;}^{\mathrm{inair}}$ or ${\mathrm{IVDT}\;}_{\mathrm{MVCT}\;}^{\mathrm{outair}}$ is closer in the chest region needs to be considered in the future.

There have been many reports on dose errors due to differences in tissue density in lungs and bones,^16^, ^17^ and this study focused on the MVCT number of air. This study shows that differences in the air MVCT numbers of IVDT can cause a dose difference of nearly 1% owing to differences in density from kVCT.

## 4 CONCLUSION

In this study, we found that the Planned Adaptive calculated doses using MVCT images were more consistent with the kVCT planning dose using ${\mathrm{IVDT}\;}_{\mathrm{MVCT}}^{\mathrm{outair}}$ than ${\mathrm{IVDT}\;}_{\mathrm{MVCT}}^{\mathrm{inair}}$ in the HN region. This study showed that ${\mathrm{IVDT}\;}_{\mathrm{MVCT}}^{\mathrm{outair}}$ was more accurate than ${\mathrm{IVDT}\;}_{\mathrm{MVCT}}^{\mathrm{inair}}$ in determining the recalculated dose of MVCT using TomoTherapy.

## AUTHOR CONTRIBUTIONS

Shuichi Ozawa and Minoru Nakao: conceptualization, methodology, software, formal analysis, review, editing, supervision, project administration, visualization, and validation.

Hideharu Miura: conceptualization, formal analysis, review, and editing.

Akito Saito and Daisuke Kawahara: formal analysis, review, and editing.

Yasuhiko Onishi, Takashi Onishi, and Taiki Hashiguchi: methodology and validation.

Yoshihisa Matsumoto and Tsutomu Maruta: methodology, supervision, review, and editing.

Yuji Murakami and Yasushi Nagata: supervision, review, and editing.

## CONFLICTS OF INTEREST

The authors declare no conflict of interest.
